# In Vitro Comparison of a Closed and Semi-closed Leaflet Design for Adult and Pediatric Transcatheter Heart Valves

**DOI:** 10.1007/s10439-024-03502-3

**Published:** 2024-04-13

**Authors:** Alexander Breitenstein-Attach, Marvin Steitz, Xiaolin Sun, Yimeng Hao, Jonathan Kiekenap, Jasper Emeis, Sugat Ratna Tuladhar, Felix Berger, Boris Schmitt

**Affiliations:** 1 Department of Pediatric Cardiology and Congenital Heart Disease, German Heart Center Berlin (Charité), Augustenburger Platz 1, Berlin, Germany; 2 Department of Pediatric Cardiology and Congenital Heart Disease, Charité — University Medicine Berlin, Augustenburger Platz 1, Berlin, Germany; 3 DZHK (German Centre for Cardiovascular Research), Potsdamer Str. 58, Berlin, Germany; 4 Department for Cardiothoracic, Transplantation and Vascular Surgery, Hannover Medical School, Carl-Neuberg-Straße 1, Hannover, Germany

**Keywords:** Congenital heart disease, Transcatheter heart valve replacement, Pulmonary valve, Valve geometry, Pinwheeling

## Abstract

Transcatheter heart valve replacements (TVR) are mostly designed in a closed position (c) with leaflets coaptating. However, recent literature suggests fabricating valves in semi-closed (sc) position to minimize pinwheeling. With about 100,000 children in need of a new pulmonary valve each year worldwide, this study evaluates both geometrical approaches in adult as well as pediatric size and condition. Three valves of each geometry were fabricated in adult (30 mm) and pediatric (15 mm) size, using porcine pericardium. To evaluate performance, the mean transvalvular pressure gradient (TPG), effective orifice area (EOA), and regurgitation fraction (RF) were determined in three different annulus geometries (circular, elliptic, and tilted). For both adult-sized valve geometries, the TPG (TPG_C_ = 2.326 ± 0.115 mmHg; TPG_SC_ = 1.848 ± 0.175 mmHg)* and EOA (EOA_C_ = 3.69 ± 0.255 cm^2^; EOA_SC_ = 3.565 ± 0.025 cm^2^)* showed no significant difference. Yet the RF as well as its fluctuation was significantly higher for valves with the closed geometry (RF_C_ = 12.657 ± 7.669 %; RF_SC_ = 8.72 ± 0.977 %)*. Recordings showed that the increased backflow was caused by pinwheeling due to a surplus of tissue material. Hydrodynamic testing of pediatric TVRs verified the semi-closed geometry being favourable. Despite the RF (RF_C_ = 7.721 ± 0.348 cm^2^; RF_SC_ = 5.172 ± 0.679 cm^2^), these valves also showed an improved opening behaviour ((TPG_C_ = 20.929 ± 0.497 cm^2^; TPG_SC_ = 15.972 ± 1.158 cm^2^); (EOA_C_ = 0.629 ± 0.017 cm^2^; EOA_SC_ = 0.731 ± 0.026 cm^2^)). Both adult and pediatric TVR with semi-closed geometry show better fluiddynamic functionality compared to valves with a closed design due to less pinwheeling. Besides improved short-term functionality, less pinwheeling potentially prevents early valve degeneration and improves durability. *Results are representatively shown for a circular annulus geometry.

## Introduction

As early as around 1950, Dwight E. Harken formulated characteristics that an optimal heart valve prosthesis must fulfil [1]. These requirements are still valid today and include permanent functionality, growth potential, absence of thrombogenesis, and immune response as well as resistance to infection. According to Harken, this is the only way to ensure the longevity of the prosthesis. In order to meet these requirements, the valve geometry plays a central role. It determines short-term functionality, i.e. adequate outflow and sufficient closure, as well as the long-term functionality, because unfavourable valve geometries lead to malformations and pathological stress loads, which are the main reason for valve calcification [2].

Despite many years of research, there is still no consensus regarding the optimal valve geometry. Most commercially available valves are designed in a closed position with leaflets coaptating. However, in 2013, Kouhi and Morsi proposed a semi-closed valve shape for transcatheter heart valve replacements (TVR) to ensure proper valve opening with low–pressure gradients [3]. They performed a parametric in silico study on geometry variations of an aortic valve and found that although the design of a heart valve in closed form ensures good tightness during diastole, this leads to poor opening behaviour during systole with a reduced opening area and higher pressure gradients. In addition, the strong curvature of the leaflet leads to unfavourable stress distributions, stress peaks and abnormal leaflet deformations during the systolic opening phase, which in turn can lead to premature failure of the valve. This approach was continued by Travaglino et al. in 2020 by conducting a computational optimization study of several TVR leaflet designs using porcine and bovine leaflets [4]. They confirmed the hypothesis of Kouhi and Morsi, stating that leaflet centre points should be close enough to the valve centre point so that the coaptation zone and, thus, the tightness are sufficient and the stress load is not concentrated [3]. At the same time, however, they should be far enough apart from each other so that they do not twist into each other when closed due to excess material, which causes a so-called pinwheeling effect. This effect verifiably leads to early tissue degeneration [5]. Although there is much research about heart valve geometries, to our knowledge, all experimental research and development on TVR including comparison of geometrical approaches have been done for adult-sized valves under corresponding testing conditions. There is no scientific proof that favourable valve designs for adults are also favourable for children. In this paper, we will compare a self-designed closed (c) with semi-closed (sc) valve geometry based on current literature first for adult-sized valve and subsequently assess the question whether these results can be transferred to pediatric valve prostheses.

## Materials and Methods

### Valve Parameterization

In this section, geometrical valve parameters are derived based on existing literature. For all mathematical expressions, the coordinate origin is located in the circumferential centre of the valve at the highest point, namely the commissures.

For a defined geometrical description, the parametrical valve design by Thubrikar was chosen and extended. From the 1980s, Thubrikar investigated human aortic heart valves and defined several geometrical parameters to describe the valve shape [6]. Labrosse et al. adopted this approach and identified two relevant regions of a closed leaflet, namely a load-bearing part with an approximately cylindrical geometry and a coaptation zone with a planar geometry [7].

**Fig. 1:**
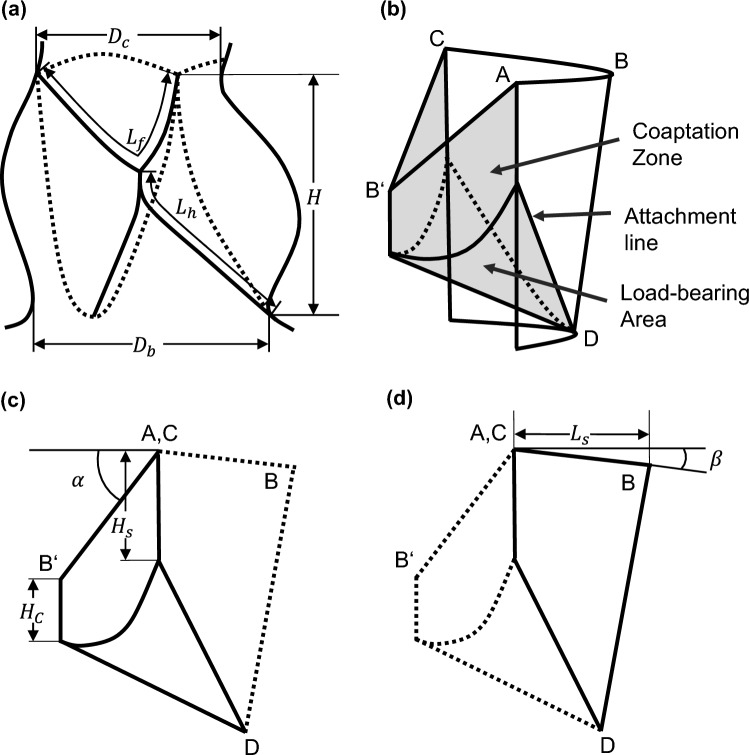
Geometrical description of the aortic valve [6–8]: **a** Aortic valve showing the side view of one leaflet with ${D}_{\text{c}}$: Commissural diameter; ${D}_{\text{b}}$: Basal diameter; $H$: Leaflet Height; ${L}_{\text{f}}$: Free edge length; ${L}_{\text{h}}$: Leaflet length; **b** Schematic showing one leaflet in both open (transparent) and closed (shaded) positions with points A and C referring to the top of commissures. Point B (resp. B’) is the middle point of the leaflet-free edge in open (resp. closed) position. D is the middle point of the leaflet attachment line. **c**–**d** Schematic showing the side view of one leaflet in both the closed (**c**)) and open (**d**)) positions with ${H}_{\text{c}}$: Coaptation height; ${H}_{\text{s}}$ Commissural height; $\alpha$ (resp. $\beta$): Angle of leaflet-free edge in closed (resp. open) position

Figure 1 shows a schematic reproduction of the geometric description of the aortic valve based on literature. Figure 1a illustrates a sectional view of the aortic valve including the surrounding vessel wall. Figure 1b, c, and d shows the lateral view of an open and a closed leaflet [6–8].

For this experimental study, the valve height $H$, which describes the total length from the annulus to the commissures, is adopted. Due to a cylindrical stent and valve clamping within pulse duplicator, ${D}_{\text{b}}$ and ${D}_{\text{c}}$ are similar and summed up to a general valve diameter $D$. In addition, the coaptation height ${H}_{\text{c}}$ was also adopted. Based on Labrosse et al., an inclination angle $\alpha$ of the free edge in regard to the commissure is introduced for this study [7]. $\alpha$ is added within this study to model the closed geometry.

**Fig. 2:**
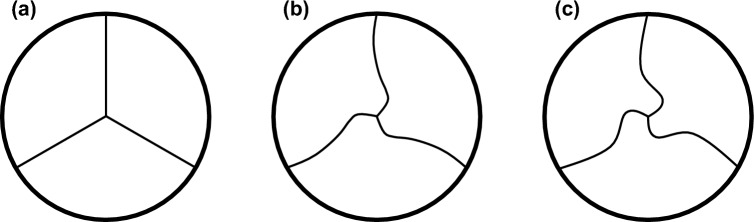
Top view of a heart valve with normal coaptation (**a**), light pinwheeling (**b**) and strong pinwheeling (**c**)

Conventionally, valves are either described in a closed or opened position [7]. An intermediate position is usually not taken into account in the geometrical description of a heart valve. Therefore, most valves are fabricated in a closed position, theoretically ensuring a proper closing behaviour. However, in 2013, Kouhi and Morsi proposed the semi-closed valve shape, which still ensures sufficient closure but reduces the transvalvular pressure gradient and also generates more favourable stress distribution with lower peak stress [3]. This approach was continued by Travaglino et al. who even proposed a better closing behaviour for the semi-closed geometry [4]. If the valve is fabricated with closed leaflets, these leaflets will inevitably twist into each other after implantation because the implantation diameter is ~ 10–20% smaller than the nominal stent size [9]. This results in a pinwheeling effect, which leads to pathological peak stress and early valve degeneration [10]. Therefore, they state that the opening degree is extremely important, because the valve shall have enough leaflet material to close properly, but not too much to create a large pinwheeling effect [4]. Figure 2 illustrates this effect by means of a top view of three closed heart valves with no (Fig. 2a), a slightly (Fig. 2b) and a moderately (Fig. 2c) pronounced pinwheeling effect.

**Fig. 3:**
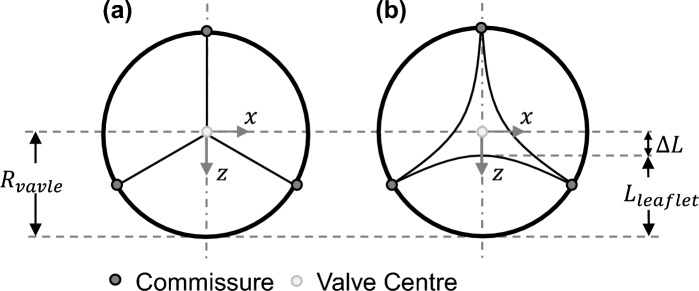
Modified leaflet parameterization for closed (**a**) and semi-closed (**b**) valve design based on Xu et al. [11]

In order to quantify this opening degree, a parameter $a$ is introduced. Following the parametric study of Xu et al., this parameter describes the distance of the leaflet tip to valve centre in percentage [11]. The parameter $a$ is mathematically formulated in formula 1. All corresponding geometrical parameters are visualized in Fig. 3a and b that displays the valve top view of the closed as well as the semi-closed valve design, respectively. ${R}_{{\text{valve}}}$ represents the valve radius, ${L}_{{\text{leaflet}}}$ the maximum length of one leaflet and $\Delta L$ the difference in length of these two parameters.

1 $$a=\frac{\Delta L}{{R}_{{\text{valve}}}}*100=\frac{{R}_{{\text{valve}}}-{L}_{{\text{leaflet}}}}{{R}_{{\text{valve}}}}*100 [\%].$$

In addition to the parameterization in the top view, the leaflet belly needs to be described mathematically in the side view. This can be done using a second-order surface. As early as 1843, Anders A. Retzius described the leaflet belly of the aortic valve as part of a sphere [12]. On the other hand, Mercer et al. describe the leaflet belly as half a paraboloid [13]. However, Jiang et al. found a sphere as well as a paraboloid to be unfavourable due to high-pressure gradients during systole [14]. Therefore, an ellipsoidal belly shape is also presented as an option in this study. Due to the morphological similarity of the aortic and the pulmonary heart valve, a transferability of these geometrical approaches to a pulmonary TVR is assumed [15, 16]. A detailed consideration of the belly shape used within this study is given in the following section.

### Valve Construction

For the quantitative description of both geometrical approaches, the previously introduced parameters are first defined for the adult-sized valves. To maintain the exact shape also in a pediatric size, the geometries are subsequently scaled down proportionally, based on the valve diameter.

Due to the fabrication process, the valve height was set to 16.75 mm. To maintain comparability between the two geometries, this parameter remains constant for both approaches. In addition, a valve diameter of 30 mm was chosen.

TVRs are always manufactured with a larger diameter compared to the post-implantation diameter to ensure proper anchoring of the stented valve. This difference in diameter is called oversizing. Considering this, a valve fabricated in a closed design will eventually show pinwheeling in the post-implantation diameter. In general, the clinically aimed oversizing value of a TVR is 10–20% but differs between commercially available TVR systems. This interval can also be exceeded for some valve areas in some patients [17] and depends among other things on the stent radial force or an intra- or supra-annular implantation position [17]. As an example, the intra-annular implantable Edwards *SAPIEN 3* TVR is approved for oversizing values ranging from 3 to 22%, while the Medtronic Melody™ TPV shall have an annular oversizing of up to 19% [18, 19]. In comparison, the supra-annular Medtronic CoreValve™ Evolut™ R system requires an oversizing of minimum 10% and maximum 26% [20]. To investigate a critical point within this study, the geometrical parameter $a$, which displays the relative distance of the leaflet tip to the valve centre (see Formula (1), is set to 30% for the semi-closed design. For the closed design, all leaflets are fully coaptating, thus, resulting in a value of 0% for parameter $a$ as seen in Fig. 3a.

Following Retzius, the load-bearing area is described through a sphere. In order to reach the valve height of 16.75 mm, an additional coaptation zone with an additional coaptation height of 1.75 mm was added. This is equivalent to the minimum coaptation height of 0.1*${R}_{{\text{valve}}}$ proposed by Thubrikar [6]. For a reproduction of the valve geometry, which is as native as possible, the free edge has an inclination angle $\alpha$ of 7° as displayed in Fig. 1 [21].

**Fig. 4:**
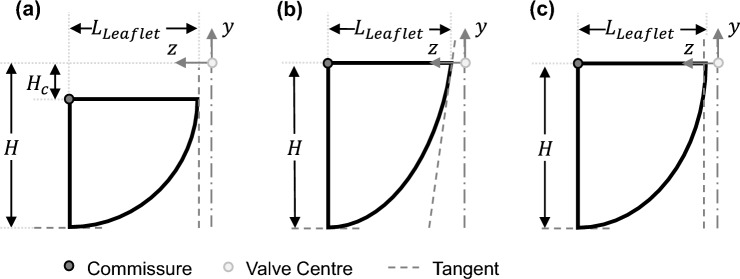
Side view of one leaflet with spherical (**a**), parabolic (**b**) and ellipsoidal (**c**) belly shape

For the semi-closed geometry, a spherical belly shape was considered unfavourable because as the parameter $a$ increases, the length of the actual leaflet decreases. In order to achieve the same valve height $H$, the initial coaptation height would need to be increased disproportionally. To remain a vertical tangent at the upper tip of the leaflet, an elliptical shape was chosen over a parabolic. For better visualization, Fig. 4a illustrates the side view of a leaflet with a spherical shape, Fig. 4b shows a leaflet with a parabolic geometry and Fig. 4c with an ellipsoidal shape. For all three leaflets, the tangents are drawn at the annulus as well as the upper leaflet tip using grey-dashed lines. Only the spherical as well as elliptical leaflets consist of similar tangent slopes.

Mathematically, the belly shape of the spherical leaflet is described through Formula 2:

2 $$\frac{{(y-{H}_{{\text{c}}})}^{2}}{{(H-{H}_{{\text{c}}})}^{2}}+\frac{{\left(z-{R}_{{\text{valve}}}\right)}^{2}}{{\left({R}_{{\text{valve}}}*\left(1-\frac{a}{100}\right)\right)}^{2}}=1.$$

For the elliptical leaflet, Formula 3 describes the belly shape.

3 $$\frac{{y}^{2}}{{H}^{2}}+\frac{{\left(z-{R}_{{\text{valve}}}\right)}^{2}}{{\left({R}_{{\text{valve}}}*\left(1-\frac{a}{100}\right)\right)}^{2}}=1.$$

As the parabolic shape is considered unfavourable, a mathematical representation of it is omitted.

Furthermore, the constructed leaflets do not have an initial coaptation zone, as this is considered contrary to the geometric approach of a semi-closed geometry. It was assumed that the valve closes due to the diastolic pressure difference and only has a coaptation zone in the closed position. ${H}_{{\text{c}}}$ is, therefore, set to 0 mm.

**Fig. 5:**
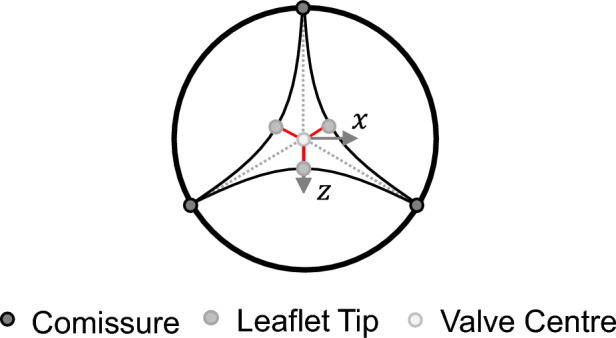
Top view of a heart valve with maximum leaflet distance to coaptation area marked in red

Since there is no initial coaptation zone and also less leaflet tissue that can potentially coapt, it is considered as unfavourable to reduce more tissue using an inclination angle. In addition, the tip of the leaflet has the longest distance to the radially symmetrical coaptation line, which is why a resulting inclination in the closed position is presumed. Figure 5 visualizes this assumption. The maximum distance of the leaflet tip to the valve centre during valve construction is marked in red (=$\Delta L)$, whereas the coaptation line is marked using a dotted grey line.

Table 1 summarizes the previously introduced geometrical parameters and shows the mathematical value of each parameter of the adult-sized valves for both geometries.

**Table 1:** Summary of design parameters for both geometrical approaches of the adult heart valve prostheses

Parameter	Closed	Semi-Closed
$H$[mm]	16.75	16.75
$D$[mm]	30	30
$a$[%]	0	30
Belly Function [-]	Sphere	Ellipsoid
${H}_{\text{c}}$[mm]	1.75	0
$\alpha$[°]	7	0

The determining parameter for the pediatric valves is diameter $D$. Here, a diameter of 15 mm was chosen. Therefore, all size parameters in Table 1 are multiplied by the factor 0.5 for the pediatric prostheses to maintain the geometrical proportions.

### Valve Fabrication

Porcine pericardium was used to fabricate the heart valves. Since an inhomogeneous thickness of the pericardium has a negative influence on the valve functionality, a uniform pericardium thickness was aimed for. Following Hiester and Sacks, the area above the left ventricle should be chosen for this [22]. Therefore, the area of the left ventricle of a pig's heart was marked, and a patch of size 9 × 9 cm was cut out. Subsequently, the pericardium was cleaned from fatty tissue and stored in a phosphate-buffered saline solution (PBS), an antibiotic combination of penicillin and streptomycin, and an antifungal agent (amphotericin B) at 4 °C for maximum two weeks.

In order to fabricate the valves, moulds were 3D printed in accordance with the valve parameters described in Table 1. Subsequently, the tissue was treated with commercially available glutaraldehyde at a concentration of 0.625 % to ensure form stability. After a two-hour exposure period, the collagen fibres were sufficiently cross-linked and the valves were washed three times by rotation in fresh PBS for 20 seconds [23]. The newly formed heart valve was then sutured into a cylindrical nitinol stent framework using the non-resorbable suture Prolene 5-0 (Ethicon Inc., Somersville/USA) in a continuous fashion.

Since pericardium is an inhomogeneous and anisotropic biomaterial, it is difficult to reproduce exact replicate valves for testing. To generate a minimum statistical relevance, three heart valve prostheses were fabricated for each geometry and size. For visualization, a photo of a closed and semi-closed TVR prior to implantation is shown in Fig. 9 within the appendix.

### Valve Test Conditions

A commercially available pulse duplicator (ViVitro Labs Inc., Victoria/Canada) was used for in vitro assessment.

Physiological saline (0.9% NaCl and distilled water) was used as test fluid. The advantage of saline is a lower temperature dependence of viscosity compared to the conventional blood-mimicking fluids. Furthermore, this test fluid is also frequently used in literature [24].

**Fig. 6:**
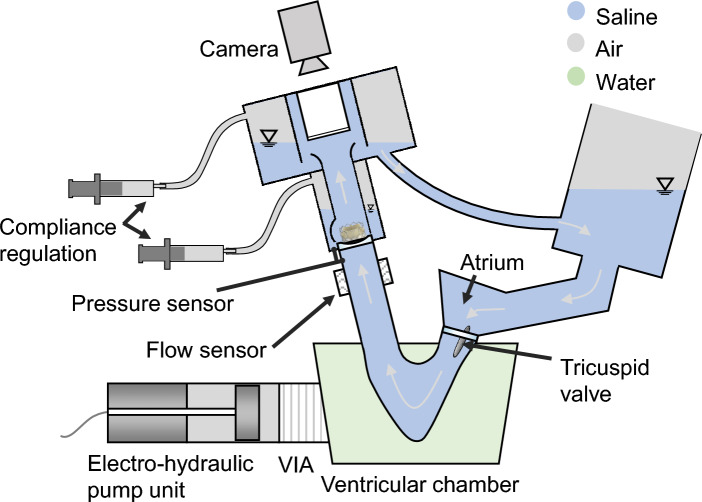
Schematic visualization of the ViVitro pulse duplicator

As approximately 70% of all pediatric heart valve diseases affect the pulmonary valve, the valves within this study are tested under pulmonary conditions [25]. To achieve the right pressure conditions, the test bench had to be modified by using a bigger compliance volume. Due to this modification, the heater was removed and a fluid temperature of room temperature (23 ± 2 °C) had to be chosen. A schematic representation of the pulse simulator is shown in Fig. 6. A labelled photo of the complete setup is shown in Fig. 10 within the appendix.

For the adult-sized valves, normotensive pulmonary pressure conditions were chosen. According to DIN EN ISO 5840-1:2021 [10], this is a right ventricle peak systolic pressure of 18–35 mmHg, a pulmonary artery end diastolic pressure of 8–15 mmHg and a peak differential pressure across the closed pulmonary valve of 13–28 mmHg [10]. Pathophysiological pressure conditions were not examined. A cardiac output of 5.0 l/min with a heart rate of 70 bpm, a MAP of 20 mmHg and a systolic time span of about 35% was aimed for. As the normotensive pressure values, these values display medium physiological flow conditions which are required for both regurgitant volume and pressure difference assessment as per DIN EN ISO 5840-3:2021 [5].

Following DIN EN ISO 5840-3:2021, three different valve holders were fabricated for the three adult-sized valves for a more realistic comparison of the two geometrical approaches [5]. The first holder consists of a simple cylindrical geometry with a diameter of 26 mm. This creates a clinically relevant oversizing of 15 % of the stented valve compared to the artificial annulus diameter.

As the native annulus is not perfectly round but oval, the influence of the elliptical clamping was examined, too. According to ISO 5840-3:2021, a major to minor axis ratio of 1.2/1.0 with a constant circumference to the circular clamping was used [5]. This corresponds to a major axis length of 28.306 mm and a minor axis length of 23.586 mm.

Due to anatomy or implantation-related conditions, a TVR is not always positioned exactly along the axis of blood flow. A prosthesis can potentially be tilted with respect to the flow axis [26]. Hatoum et al. found reduced valve functionality for tilted prostheses [26]. Therefore, the prosthesis must have sufficient fluid dynamic functionality even in a slightly tilted position. In order to determine valve functionality under malpositioning, the valve holder was modified in accordance with the challenge tests described in ISO 5840-3:2021, so that the heart valve has a tilting angle of 5° in regard to the blood flow [5]. Since an elliptical annulus cross section is more native like and a comparability to the 0° tilt was aimed to be established, an elliptical shape with the same major and minor axis lengths as in the previously presented clamping was chosen. The minor axis was used as the axis of rotation for this tilt.

To investigate the transferability of the results for the adult-sized prostheses to pediatric valves, a cylindrical holder with a diameter of 13 mm was used to maintain the 15 % oversizing. Based on the body surface area, this corresponds approximately to a one year old [27, 28]. According to ISO 5840-1:2021, this is a toddler by definition [10]. Thus, a systolic duration of 45 %, a MAP of 20 mmHg, a heart rate of 100 bpm, and a CO of 3.0 l/min were aimed for. Analogous to the previous test conditions of the adult population, the normative values were chosen for heart rate and CO. Pathophysiological circumstances were not investigated.

As required in the ISO 5840-1:2021 for verification of the pulse duplicator functionality, the commercially available TVR Acurate neo serves as a control valve and is also assessed [10].

### Valve Test Parameters

In order to comply with ISO 5840-3:2021, 10 consecutive cycles are captured for each valve. The following parameters are measured for each cycle: [5]

- Simulated cardiac output
- Cycle rate
- Systolic duration
- Forward flow volume
- Mean and RMS flow rates
- Mean pressure difference
- Effective orifice area
- Regurgitant volume, closing volume and leakage volume
- Mean arterial pressure over the whole cycle
- Appropriate qualitative photographic documentation

For evaluating the valve performance, the transvalvular mean pressure gradient (TPG) and effective orifice area (EOA) are used to describe the valve-opening behaviour, whereas the regurgitation fraction (RF) is assessed for the closing behaviour. The TPG is the time-averaged arithmetic mean value of the pressure difference across a heart valve prosthesis during the positive differential pressure period of the cycle. As per ISO 5840-1:2021, the positive differential pressure period is the period when ventricular pressure is higher than the arterial [10]. The EOA is the valve’s “orifice area that has been derived from flow and pressure or velocity data” [10]:

$$EOA=\frac{{q}_{{\text{v}}_{\text{RMS}}}}{51.6*\sqrt{\frac{TPG}{\rho }}},$$

${q}_{{\text{v}}_{\text{RMS}}}$ is the root-mean-square forward flow (ml/s) during the positive differential pressure period, which is calculated with

$${q}_{{\text{v}}_{\text{RMS}}}=\sqrt{\frac{{\int }_{{t}_{1}}^{{t}_{2}}{q}_{v}{(t)}^{2}dt}{{t}_{2}-{t}_{1}}}$$

${q}_{\text{v}}(t)$ is the instantaneous flow at time $t$, where ${t}_{1}$ is time at start and ${t}_{2}$ is time at end of positive differential pressure period, respectively.

To derive the RF, the ratio of regurgitant volume and the forward flow volume is calculated. The regurgitant volume is the sum of the closing and the leakage volume.

To evaluate valve performance, a mean value for each of the fluiddynamic testing parameters is calculated from all three valves of each group (closed and semi-closed; adult and pediatric). The resulting standard deviation displays the fluctuation within each group and test parameter.

## Results

### Hydrodynamic Parameters

Table 2 sums up the three testing parameters for the closed (c) and semi-closed (sc) adult-sized valves in circular, elliptical, and tilted annulus geometry, respectively. The following values display the mean values along with the standard deviation.

**Table 2:** Measured hydrodynamic parameters for the adult-sized valves

	TPG [mmHg]	EOA [cm^2^]	RF [%]
Circular (c)	2.33 ± 0.12	3.69 ± 0.26	12.66 ± 7.67
Circular (sc)	1.98 ± 0.18	3.58 ± 0.03	8.72 ± 0.98
Elliptic (c)	1.79 ± 0.20	3.72 ± 0.22	20.23 ± 9.90
Elliptic (sc)	2.00 ± 0.19	3.55 ± 0.17	8.94 ± 1.56
Tilted (c)	1.97 ± 0.16	3.94 ± 0.32	22.72 ± 4.43
Tilted (sc)	2.16 ± 0.31	3.70 ± 0.27	10.02 ± 1.29

Data were presented as mean ± standard deviation.

Regarding the opening behaviour of the adult valves, no significant difference was seen. All valves show a transvalvular pressure gradient as well as effective orifice area in accordance with the ISO 5840-3:2021. [5]

For the regurgitation fraction, the changes in geometry show a significant influence by utilizing an unpaired two-tailed *t* test (*p* = 0.0034). For all three testing conditions, the prostheses with the closed geometry have a higher insufficiency. Already in circular clamping, the RF_c_ is 45% higher compared to RF_sc_ design in relative terms. This ratio increases as the holder circumferences change to an elliptic shape. Here, the RF_c_ is 123% higher than RF_sc_, relatively. With an absolute RF_c_ value of 20.23% in the elliptic and 22% in the tilted position, the closed valve design exceeds the maximum threshold of 20%, which is defined by the ISO 5840-3:2021 [5].

Besides the absolute RF values, the corresponding standard deviations between both valve designs differ as well. Prostheses with the closed geometry show significantly higher deviations in all three testing conditions compared to the valves with the semi-closed design, shown by an *F* test (*p* < 0.0001).

The control valve Acurate neo showed a RF of 2.34%, a TPG of 2.2 mmHg and an EOA of 2.42 cm^2^.

Table 3 summarizes the results for the pediatric heart valves in circular annulus circumference.

**Table 3:** Measured hydrodynamic parameters for the pediatric valves

	TPG [mmHg]	EOA [cm^2^]	RF [%]
Closed geometry	20.93 ± 0.5	0.63 ± 0.02	7.72 ± 0.35
Semi-closed geometry	15.97 ± 1.16	0.73 ± 0.03	5.17 ± 0.68

Data were presented as mean & ± standard deviation

### Leaflet Kinematics

**Fig. 7:**
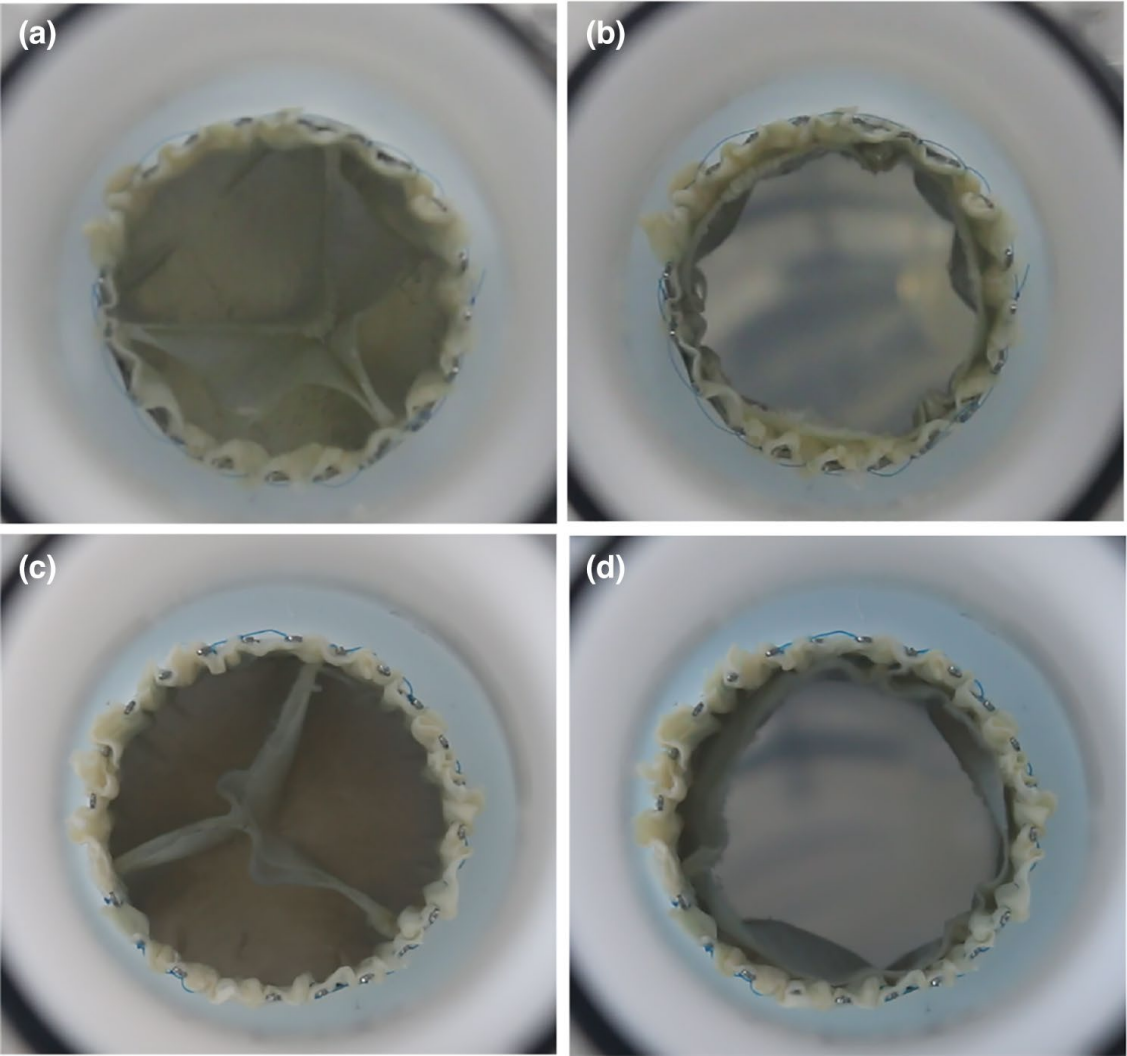
Videographic recordings of the closed (**a**, **b**) and semi-closed (**c**, **d**) adult-sized heart valves during diastole and systole in circular annulus, respectively

Leaflet kinematics were captured qualitatively using videographic recordings of the prostheses from the top view as shown in Fig. 6. Analogous to the quantitative data, no difference was seen for the valve opening behaviour during systole. The significant difference in RF as well as its standard deviation were also evident in the recordings. Prostheses with the closed design showed less uniform valve closing with increased pinwheeling compared to the semi-closed valves. Figure 7 shows representative images during diastole and systole of closed (Fig. 7a, b) and semi-closed (Fig. 7c, d) prostheses in a circular annulus. Prostheses in the elliptic as well as tilted annulus are illustrated in Figs. 11 and 12 within the appendix, respectively. Video recordings of the representative heart valve prostheses are found in the online supplementary material.

**Fig. 8:**
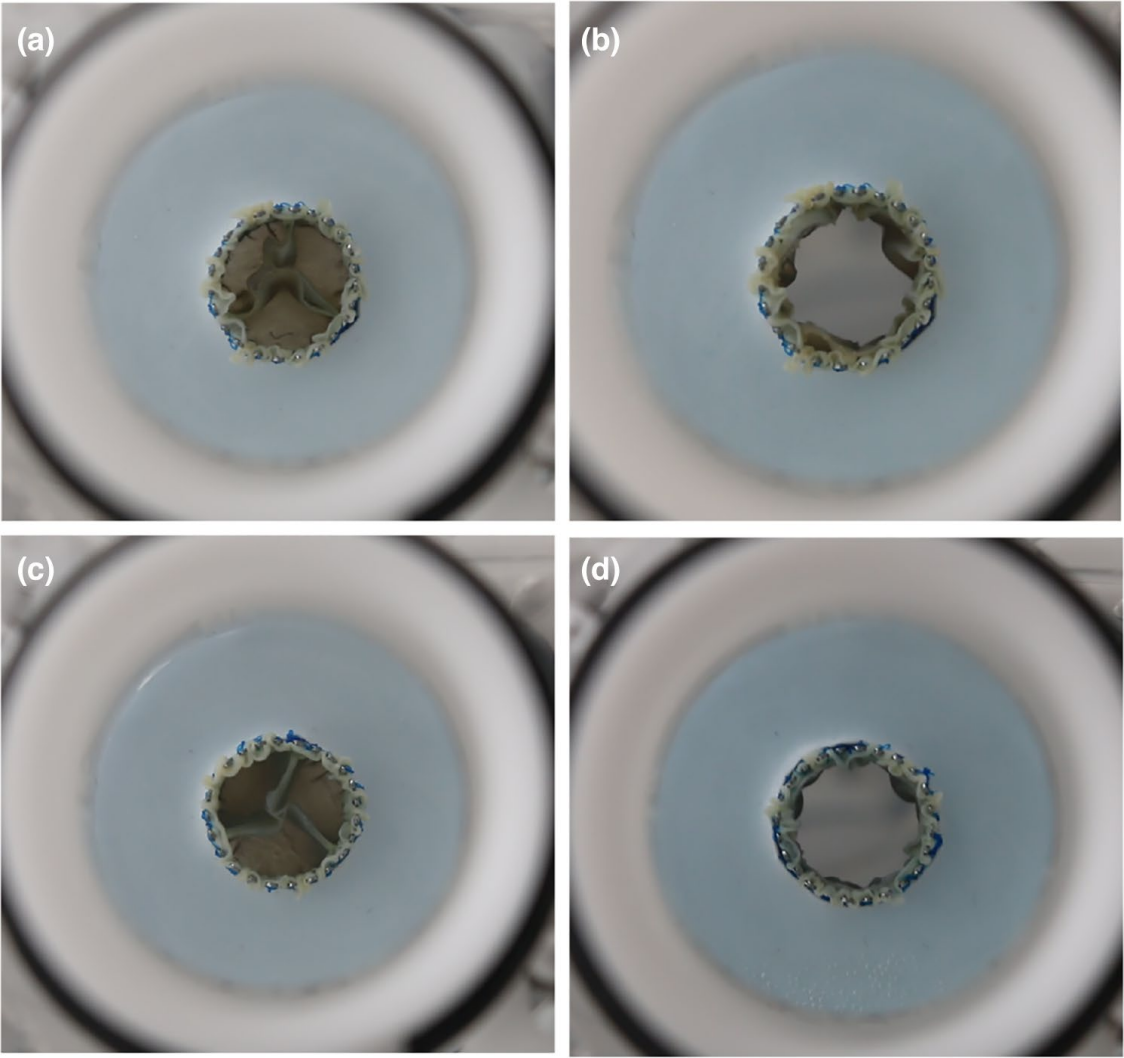
Videographic recordings of the closed (**a**, **b**) and semi-closed (**c**, **d**) pediatric heart valves during diastole and systole, respectively

**Fig. 9:**
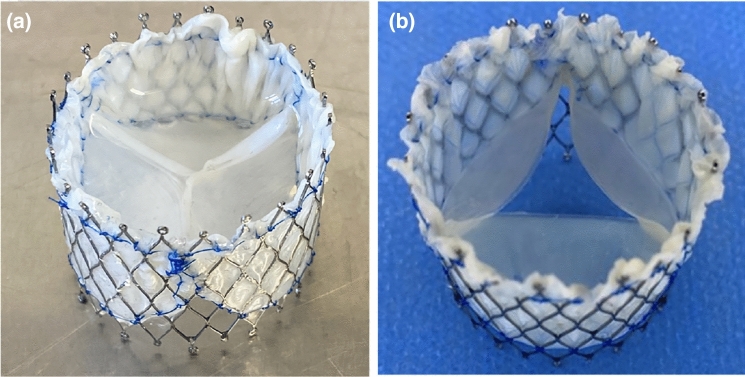
Manufactured prosthesis with **a** closed and **b** semi-closed design prior to implantation

**Fig. 10:**
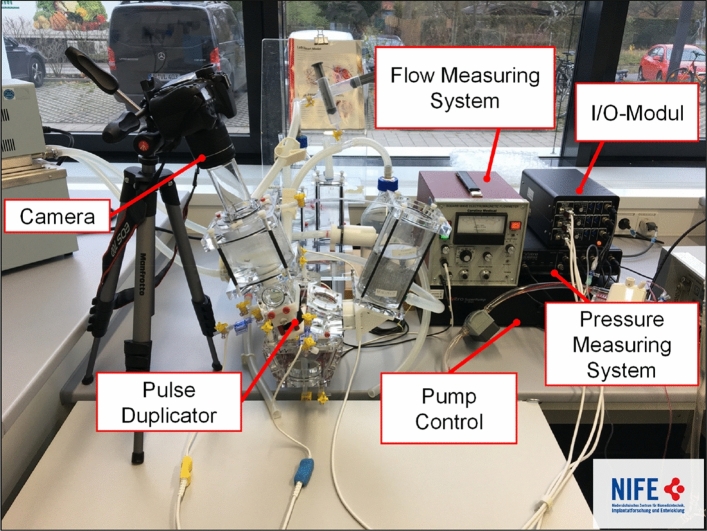
Labelled pulse duplicator test setup after modification to right heart system

**Fig. 11:**
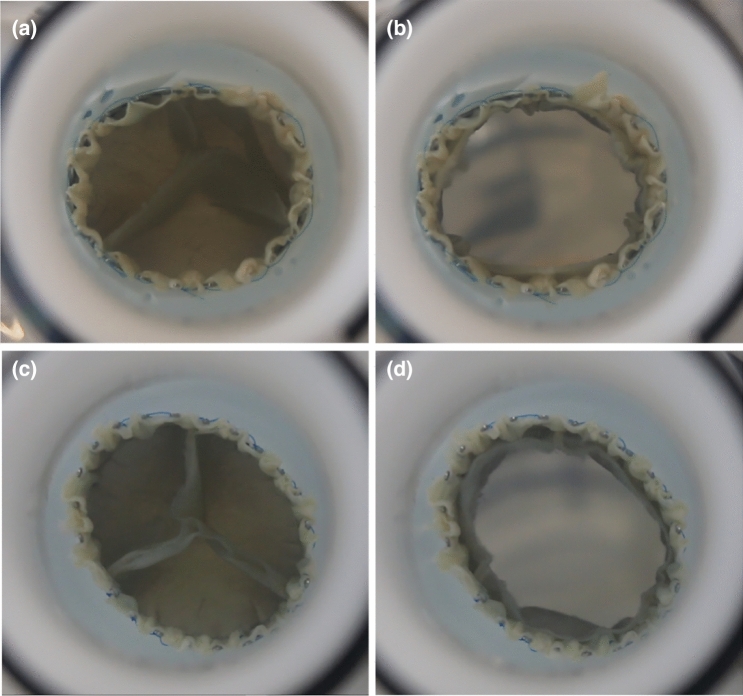
Videographic recordings of the closed (**a**, **b**) and semi-closed (**c**, **d**) adult-sized heart valves during diastole and systole in elliptic annulus, respectively

**Fig. 12:**
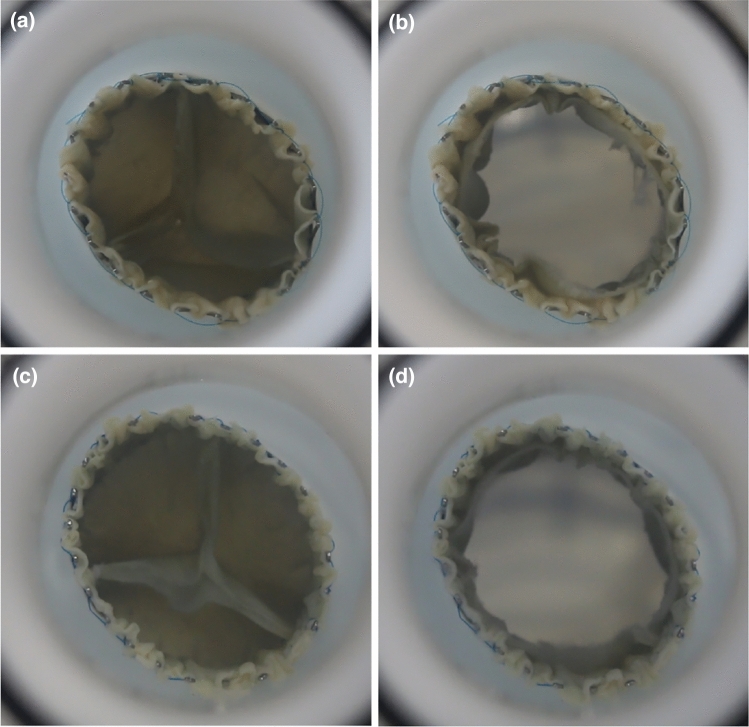
Videographic recordings of the closed (**a**, **b**) and semi-closed (**c**, **d**) adult-sized heart valves during diastole and systole in tilted annulus, respectively

As for the results of the adult valves, the RF of the pediatric prostheses with a semi-closed geometry is 33% reduced, relatively. While the semi-closed prostheses show a coaptation with little pinwheeling, the prostheses with a closed design show a coaptation line below the actual free edge and, therefore, a higher degree of pinwheeling. This is shown in Fig. 8.

## Discussion

In general, the absolute values of the control valve verified the proper functionality of the utilized pulse duplicator [29]. All aimed normotensive pressure and medium physiological flow values were achieved within the tolerance interval and can, therefore, be considered as quantitatively valid.

For both the adult and the pediatric prostheses, valves with the closed geometry had significantly reduced tightness with more inhomogeneity and pinwheeling compared to the semi-closed valves. In the circular and elliptic annulus, one adult-size TVR is above the RF limit of 20% set by DIN EN ISO 5840-1:2021 and, therefore, considered as not functional [10]. In the tilted annulus, two valves exceeded that threshold. In comparison, none of the adult-sized semi-closed valves showed RF higher than 20%. This confirms the beneficial valve functionality of semi-closed valves proposed by Kouhi and Morsi [3]. A possible explanation for the higher insufficiency of valves with closed design is a surplus of tissue material. The closed geometry was designed with an initial coaptation area in a closed state to create adequate closure at that nominal valve diameter. Since valves are implanted with a diameter below the nominal valve diameter because of oversizing, this results in a surplus of tissue material at valve closure after implantation. This surplus prevents a straight coaptation line and causes twisting of the leaflets into each other, which leads to the negative pinwheeling effect as described by Travaglino et al. [4]. Recorded videos verify this explanation. Not only twist the leaflets of the closed design into each other and cause pinwheeling but also close underneath the actual free edge line as visualized in Fig. 7a. Therefore, there is extra tissue material above the actual coaptation line, which does not contribute to the valve closure. This further surplus of material potentially prevents the leaflets from adequate coaptation and consequently reduces the sufficiency of the entire valve. This is also a potential reason for the significant difference in standard deviation. During some heart cycles, the valve closes sufficiently and in other heart cycles, the extra tissue blocks the other leaflets and causes that leakage. Yet further increasing $a$ and, therefore, decreasing the actual leaflet tissue material could potentially reduce the remaining pinwheeling. The negative influence of a tilt of the prostheses on its functionality, stated by Hatoum et al., could also be confirmed based on the derived results within this study [26].

Concerning the TPG and EOA, which were used to describe the valve-opening behaviour quantitatively, no significant difference was found for the adult-sized TVRs. All valves did not exceed the pathophysiological TPG of 12 mmHg of European Society of Cardiology [30] .Also, each TVR did not surpass the EOA threshold of 2.1 cm^2^, defined by DIN EN ISO 5840-3:2021 [5]. Based on the results of the adult valves, a comparable opening behaviour for both pediatric geometries was assumed. Contrary to this assumption, the results of the pediatric heart valves show an improved opening for the semi-closed prostheses. The transvalvular pressure gradient is lower and the effective opening area is larger. In general, the TPG is significantly larger for both geometries compared the adult-sized valves. One possible explanation is the size of the annulus because the pressure gradient increases as the valve size decreases [31]. Compared to the adult heart valves, the gradients of the pediatric valves increased 9-fold for the closed and 8-fold for the semi-closed design. Hence, the gradient increases more for the closed than the semi-closed geometry. In addition, the semi-closed valves already have a smaller gradient in the circular testing condition for adults. This previously existing difference is then amplified by this gradient increase and the discrepancy becomes more pronounced. This has a direct effect on the EOA. According to the mathematical relationship of TPG and EOA, the EOA decreases due to an increased gradient at a constant CO. This is also visible from the measured values. Thus, the negative influence described by Jiang et al. due to the spherical belly area is recognizable [14].

Besides the difference between both pediatric valve designs, the significant TPG increase for both designs compared to the adult TVRs is conspicuous and cannot be explained physiologically solely by a smaller valve and annulus diameter. Generally, the TPG increases as the valve diameter decreases. Permanyer et al. reported a regressive increase of 1 mmHg as the diameter decreases 2 mm. However, the increase of both gradients is significantly higher compared to the trend Permanyer et al. presented. One possible explanation is the pulse duplicator system itself as it is made for adults. Therefore, we are facing a too large ventricle and artery for pediatric investigations, while the valve and the annulus is accordingly small. Fluiddynamically, as the ventricle ejects the fluid, the fluid faces a sudden and non-anatomical cross-sectional narrowing at the valve, which causes non-physiological vortices, turbulences, flow separations and other negative effects and is further assessed in the following section. Another explanation is the tissue material as it is harvested from adult pigs with a greater thickness than babies or adolescents. As the diameter decreases, but the leaflet thickness remains the same, the mass inertia and stiffness is potentially too high, resulting a too high resistance to valve opening and non-physiological TPG. Future studies should assess the influence of tissue thickness on the fluiddynamic parameters.For both adult and pediatric heart valve prostheses, the advantages of the semi-closed design are evident. Therefore, based on the results generated within this study, a transferability of beneficial valve designs from adult to pediatric prostheses has been successfully confirmed in vitro. Future studies should investigate more variations of the introduced opening degree $a$ which could reduce pinwheeling even further. However, it should be noted that this geometrical study has been performed on our own valve designs that are derived based on the current literature. Therefore, the study is valid for these designs. As the idea of shaping a self-expandable TVR in a semi-closed state is a general approach and also published by other research groups, a transferability to other TVRs utilizing a self-expandable stent is potentially possible. For example, existing commercial TVRs with a closed design, such as the Medtronic *CoreValve™ Evolut™ R*, which requires a minimum oversizing of 10 % as per instruction for use, could potentially benefit from a semi-closed geometry in terms of reduced pinwheeling and slower degeneration [20].

### Limitations

This study has several limitations that need to be assessed. One limitation is the valve material. Since it is a biological material, the fibre structure and material thickness of each heart valve is different. This can potentially lead to a reduced thickness of one leaflet compared to the other two. As a result, the individual leaflets do not close uniformly and may impair valve functionality. Although the tissue above the left ventricle was used for all prototypes, neither fibre structure nor homogeneity of thickness could be checked quantitatively. Solely a visual inspection was carried out.

Another limitation is the test bench in regard to the verification of the pediatric heart valves. The test bench is designed for adult valves. For this reason, the ventricle as well as the arterial vessel diameter is disproportionately large. Thus, the pediatric heart valve represents a non-anatomical constriction and a non-physiological flow profile occurs. This may lead to pathological flow velocities, turbulence or further energy loss. It is, therefore, possible that the opening or closing behaviour of the pediatric valves was affected by the fluid dynamics. Therefore, absolute measured values may be distorted or potential fluctuations are not detected. However, since all prostheses experienced the same test conditions, validity of the verification is assumed.

Following from that limitation, both the adult-sized and pediatric TVRs were tested in circular annulus shapes that are required as per DIN EN ISO 5840-3:2021 [5]. However, the anatomically more realistic oval annulus shape was solely assessed for the adult-sized valves. Although the transferability from beneficial adult-sized to pediatric TVR geometry was shown normatively in a circular annulus, the complete transferability should be assessed for oval and tilted annulus as well within future studies.

## Appendix

See Figs. 9, 10, 11 and 12.
